# Recent advances in the surgical management of rhinosinusitis

**DOI:** 10.12688/f1000research.9163.1

**Published:** 2016-09-26

**Authors:** Alexandria F. Jaksha, Erik K. Weitzel, Adrienne M. Laury

**Affiliations:** 1 Uniformed Services University of the Health Sciences, Bethesda, MD, 20814, USA; 2 Department of Otolaryngology–Head and Neck Surgery, San Antonio Uniformed Services Health Education Consortium (SAUSHEC), San Antonio Military Medical Center, Houston, TX, 78234, USA

**Keywords:** chronic rhinosinusitis, nasal polyps, surgical management, sinus surgery

## Abstract

Rhinosinusitis affects a significant portion of the US population, and its management imposes a substantial burden on the healthcare system. The treatment of chronic rhinosinusitis includes initial medical management prior to consideration of surgical intervention. However, if surgery does become necessary, several factors must be considered in order to optimize outcomes. This review evaluates surgical patient selection, perioperative medical management, and the extent of operative intervention, with the goal of improving surgical results, decreasing the need for revision surgery, and enhancing the patient’s quality of life. Specific variations in patient genotypes and phenotypes will be further explored with regard to their implications on surgical outcomes. Additionally, the evidence behind pre- and post-operative antibiotic and steroid use will be evaluated. Finally, we will review evolving surgical tools and techniques that are currently being utilized for the treatment of specific subsets of rhinosinusitis.

## Introduction

Rhinosinusitis is a relatively common disease affecting approximately 13% of the US population and costing healthcare systems more than $8.6 billion annually ^1, 2^. Treatment usually commences with medical management, including nasal saline irrigations, topical nasal steroids, oral antibiotics, and possibly oral steroids ^3^. However, medical management oftentimes fails to sufficiently alleviate the patient’s symptoms, and, for these patients, surgical intervention may be considered to be a logical next step in their treatment algorithm. Surgical management of rhinosinusitis is typically reserved for two main subcategories: chronic rhinosinusitis (CRS) and recurrent acute rhinosinusitis (RARS). CRS is defined by the American Academy of Otolaryngology – Head and Neck Surgery as 12 weeks, or longer, of two of a defined group of symptoms/signs: mucopurulent drainage, nasal congestion, facial pressure/pain, or decreased sense of smell AND inflammation documented by one of the following: purulent mucus on endoscopy, polyps in the nasal cavity, or radiographic imaging showing inflammation ^3^. Alternatively, RARS is diagnosed when four or more episodes of acute bacterial rhinosinusitis (<4 weeks in duration) occur in a period of 12 months without signs of rhinosinusitis between episodes ^3^. Over the past few decades, surgical intervention for these disease states has been extensively evaluated in its ability to achieve “successful outcomes” measured by endoscopic improvement, symptom reduction, and quality of life changes ^4– 10^. Additionally, Smith *et al*. showed that in patients who failed medical management, subsequent sinus surgery resulted in less antibiotic use, less steroid use, significant improvement in quality of life, and fewer missed days of work/school compared to continued medical management ^4^. Therefore, this review will focus on the various factors which must be considered in order to optimize surgical results, including patient selection, perioperative medical management, and intraoperative surgical techniques and tools.

## Surgical patient selection

In sinus surgery, optimizing patient selection is essential to achieving successful surgical outcomes. Over the past decade, patients with CRS have been frequently subdivided into two main categories based on their specific pathophysiology: CRS with nasal polyps (CRSwNP) vs. CRS without nasal polyps (CRSsNP). This segregation is based on significant differences in both genotype and phenotype. Molecularly, CRSwNP appears to be a largely T helper cell type 2 (Th2)-mediated disease process based on an upregulation of interleukin (IL)-5, eosinophils, and mast cells, while CRSsNP is usually Th1 mediated and includes an upregulation in interferon gamma and IL-8 ^11^. Clinically, CRSwNP trends towards worse pre-operative quality of life scores and symptom scores when compared with CRSsNP ^12, 13^.

Additionally, when separating patients based on the presence or absence of polyps, the implications of surgical intervention also varies. SNOT-22 (sinonasal outcome test) scores, a validated outcome measure used to assess the severity of a patient’s rhinosinusitis symptoms, have been shown to be pre-operatively worse in CRSwNP than in CRSsNP ^5, 12– 14^. However, CRSwNP patients have also been noted to have greater improvements in SNOT-22 scores after surgical intervention compared to CRSsNP patients ^12, 13^. Additionally, in CRSwNP patients, a multi-center study showed significant improvement in post-functional endoscopic sinus surgery (FESS) quality of life scores when compared to CRSwNP patients who continued medical management ^4^.

When analyzing the various subtypes of CRSwNP, specific parameters have also been linked with increased surgical success. For example, idiopathic polyps are often associated with improved surgical response when compared to polyps associated with a systemic process, such as asthma or aspirin intolerance ^15^. Eosinophilia, which has been classically associated with CRSwNP, has also shown a propensity for worse surgical outcomes based on the increased rates of polyp recurrence after surgery ^16, 17^. This may be related to the decrease in cilia along sinonasal mucosa in this subgroup ^18^. However, eosinophilia is not inherent to all CRSwNP, as exemplified by the nearly 80% of Asian CRSwNP patients whose polyps tend to be more neutrophil dominant ^6, 11, 19– 21^. Therefore, there are numerous factors within the CRSwNP subclassification that may affect the likelihood of surgical success or disease recurrence ^22, 23^.

Another unique subcategory of CRSwNP is cystic fibrosis (CF) patients. These patients are often considered “poor responders” to FESS owing to their high surgical revision rates ^24^. However, this is secondary to the underlying pathophysiology of CF, which results in ongoing sinonasal ciliary dysfunction and, in turn, chronically thick and stagnant mucus. However, post-FESS, these patients have been shown to have significant improvements in quality of life and endoscopy scores equivalent to non-CF CRSwNP controls ^25^. Additionally, revision surgeries often have similar symptom improvements and patient outcomes compared to the initial surgery ^5^. Furthermore, FESS is thought to assist in the reduction of CF flares by expunging one of the bacterial reservoirs of *Pseudomonas aeruginosa* ^26^. Therefore, while CF patients with CRSwNP are often considered to be surgical “failures”, secondary to their high revision rates, surgical intervention is still recommended based on its continued ability to improve sinonasal symptom scores as well as overall pulmonary function ^25, 27^.

Alternatively, CRSsNP patients tend to have better pre-surgical SNOT-22 scores and less relative improvement than do CRSwNP patients after FESS ^12, 13^. A large Cochrane meta-analysis showed no difference in medical vs. surgical management of CRSwNP ^28^; however, additional randomized controlled trials have shown significant improvement with FESS if patients had previously failed medical treatment ^4, 29– 34^. A recent study by Lind *et al*. showed a >50% reduction in SNOT-22 scores and a significant improvement in olfactory function at up to 6 months post-surgery for both CRSwNP and CRSsNP patients ^30^. Interestingly, in 2016, gene variations in the TAS2R83 receptor were found to be associated with a poor response to surgery in certain CRSsNP patients; however, no such correlation was noted for CRSwNP patients ^8, 35^. These findings suggest that, through advances in genome sequencing, we may be able to pre-operatively genetically evaluate surgical candidates and determine their probability of successful surgical intervention ^8^. In the meantime, while there is not a clear consensus on which CRSsNP patients will definitively benefit from FESS, it should still be considered a useful treatment option in those who have failed medical management ^5, 6, 8^.

Finally, regardless of polyp status, recent studies have evaluated the utility of cluster analysis of CRS patients and their potential in predicting surgical success. In 2016, Tomassen *et al*. showed that multiple inflammatory endotypes of CRS exist based on cluster analysis of tissue biomarkers such as IL-5 and tumor necrosis factor-α ^36^. They found that these clusters of endotypes were largely correlated with phenotypes but further differentiated them based on the inflammatory mechanisms involved. Therefore, these endotypes may be of importance for predicting comorbidities such as asthma as well as predicting the probability of disease recurrence after sinus surgery ^37^. Additionally, Soler *et al*. also reported a cluster analysis of 103 clinical variables encompassing demographics, comorbidities, objective CRS metrics, and patient outcome measures, which enabled them to identify specific patient clusters who had improved SNOT-22 outcomes with surgical intervention ^23^. This result was sustained for up to 18 months post-surgery. Interestingly, when simplified, three main variables – lost productivity, patient age, and baseline SNOT-22 – were able to accurately cluster patients and, in turn, provide prognostic information regarding the success of surgical intervention. Overall, while polyp status does appear to impact the likelihood of surgical success, several other factors such as co-morbidities, inflammatory biomarkers, patient demographics, and even genetic variations also appear to influence the efficacy of surgical intervention.

## Perioperative medical management

Perioperative management can include a number of treatment modalities, but two highly debated topics include steroids and antibiotics. Currently, providers often choose perioperative medications based on preference because studies are often limited, contradictory, or insufficient in evaluating medication utilization for specific patient phenotypes.

Pre-operative systemic steroids have been shown to reduce inflammation, polyp size, operating time, and bleeding during surgery and allow better visualization in CRSwNP patients ^38– 41^. While they have been shown to improve intraoperative conditions, pre-operative systemic steroids have not been shown to affect polyp recurrence rates or improve patient quality of life scores post-surgery ^38, 39, 41, 42^. Pre-operative topical (intranasal) steroids in CRSsNP also show intraoperative improvements ^8^. While studies found that pre-operative topical steroids lack a direct improvement in symptom scores, they do result in decreased bleeding and shorter operative times that have been correlated to improvements in symptom scores ^8^. Therefore, in CRSsNP, topical steroids appear to have comparable effects on intraoperative outcomes but without the systemic side effects of oral steroids ^5, 41^. Overall, most experts agree that pre-operative use of oral and/or topical steroids in CRSwNP and topical steroids in CRSsNP improves surgical conditions and should be considered prior to FESS ^8, 39, 41^.

Post-operative topical steroids also have been shown to play a beneficial role in improving surgical outcomes. Commonly utilized topical steroids include nasal sprays such as fluticasone or mometasone as well as budesonide respules, which can be placed into nasal saline irrigations. Multiple randomized, placebo-controlled clinical trials have shown significant improvement in clinical outcomes post-FESS when topical nasal steroids were utilized ^43– 45^. Specifically, for patients with CRSwNP, recurrence rates were reduced and length to recurrence increased ^45^. Additionally, as mentioned above, the risk for systemic side effects from topical steroids is extremely minimal. Currently, for CRS, experts recommend initiation of topical steroids approximately 2–6 weeks post-FESS in order to optimize clinical outcomes.

Alternatively, systemic steroids, secondary to their side effect profile, are often more judiciously administered post-sinus surgery ^10^. Some experts propose that they should be limited to patients with severe disease or those at high risk for recurrence ^5, 46, 47^. However, others suggest that most CRSwNP patients should receive a short post-operative course of systemic steroids to decrease the initial inflammatory response post-surgery ^48^. This is then often followed by a long-term utilization of topical steroids. Post-operative systemic steroids are also considered in CRSwNP patients, as they have been shown to improve endoscopy scores, which, in turn, can ease post-operative debridements and enhance continued medical management ^41, 46, 47^. Additionally, while most studies have focused on the use of post-operative systemic steroids in CRSwNP patients, CRSsNP patients have also shown some benefit in prospective and retrospective studies specifically with regard to endoscopy and symptom scores ^41, 46, 49, 50^. Overall, post-operative steroids, both topical and systemic, have been shown to improve endoscopy scores and symptom scores and decrease polyp recurrence and, therefore, should be considered as an adjunct in certain post-surgical patient populations ^8, 41, 49, 50^.

There are relatively few studies that examine antibiotic administration prior to FESS in CRS or RARS patients ^8^. Currently, pre-operative antibiotic use is limited to the treatment of an acute infection prior to surgery, with the goal of reducing inflammation and thereby improving the surgical field ^8^. Alternatively, one study did show that pre-operative doxycycline in CRSwNP resulted in a small decrease in pre-operative polyp size, nasal secretions, and inflammatory markers yet had no effect on quality of life metrics or surgical success ^5, 51^. Therefore, it is currently recommended that pre-operative antibiotics are indicated only in the presence of an acute infection prior to surgery ^5^.

Alternatively, post-operative oral antibiotics are traditionally continued for 7 to 10 days following surgery; however, the evidence behind this practice is limited. Macrolides, the most extensively evaluated antibiotic class in the treatment of CRS, have shown improvements in endoscopy scores, with CRSsNP having a more robust response than CRSwNP ^52^. Additionally, a recent double-blinded, placebo-controlled trial by Albu *et al*. examined a 14-day course of Augmentin post-FESS. They found an improvement in patient symptoms at 5 days and endoscopic appearance at 12 days ^53^. Post-operative antibiotics also show reproducible effects in specific patient subcategories such as CF. Because CF patients’ exacerbations are often related to bacterial colonization of the unified airway, antibiotics are often essential to the treatment of both pulmonary and sinonasal flares. Specifically, in the post-FESS period, CF patients saw symptom improvement with sinonasal inhalational tobramycin ^54^. This marks one of the only patient groups in whom topical antibiotics play a defined role in improving patient outcomes post-FESS ^54^. Overall, in most cases of CRS, experts agree that a 7–14-day course of post-operative oral antibiotics may optimize early clinical outcomes and improve endoscopy post-FESS ^8, 46^.

## Intraoperative management: techniques and tools

The extent of surgical intervention in the treatment of CRS and RARS is widely varied and debated. However, over the past decade, otolaryngologists have trended towards a more customized surgical approach for each patient based more on their disease phenotype and co-morbidities. Again, differentiating between CRSwNP and CRSsNP subtypes vs. RARS often plays a role in determining the extent of surgical management.

**Figure 1.**
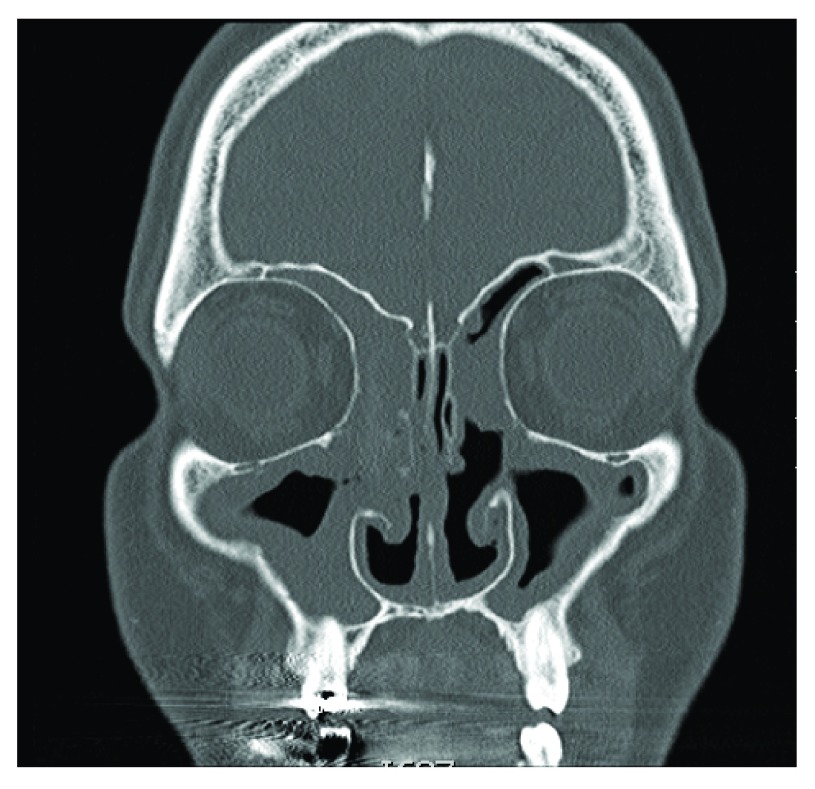
Chronic rhinosinusitis with nasal polyps (CRSwNP). Coronal CT scan of CRSwNP showing extensive polyposis lining the bilateral maxillary, ethmoid, and frontal sinuses.

Specifically, for CRSwNP ( Figure 1), surgeons often prefer a more extensive initial surgery, including widely opening all eight sinuses with the main variation being in the extent of frontal sinus intervention. This more aggressive approach is based on the underlying inflammatory process inherent in most patients with CRSwNP. Therefore, the goals of surgical management in CRSwNP are not only to remove the diseased tissue but more so to improve sinus drainage, expose more tissue for topical drug delivery, and decrease the inflammatory load ^8^. Generally, this extensive approach is more effective in improving symptom scores and reducing recurrence rates compared with more minimally invasive techniques ^5, 7– 9^.

**Figure 2.**
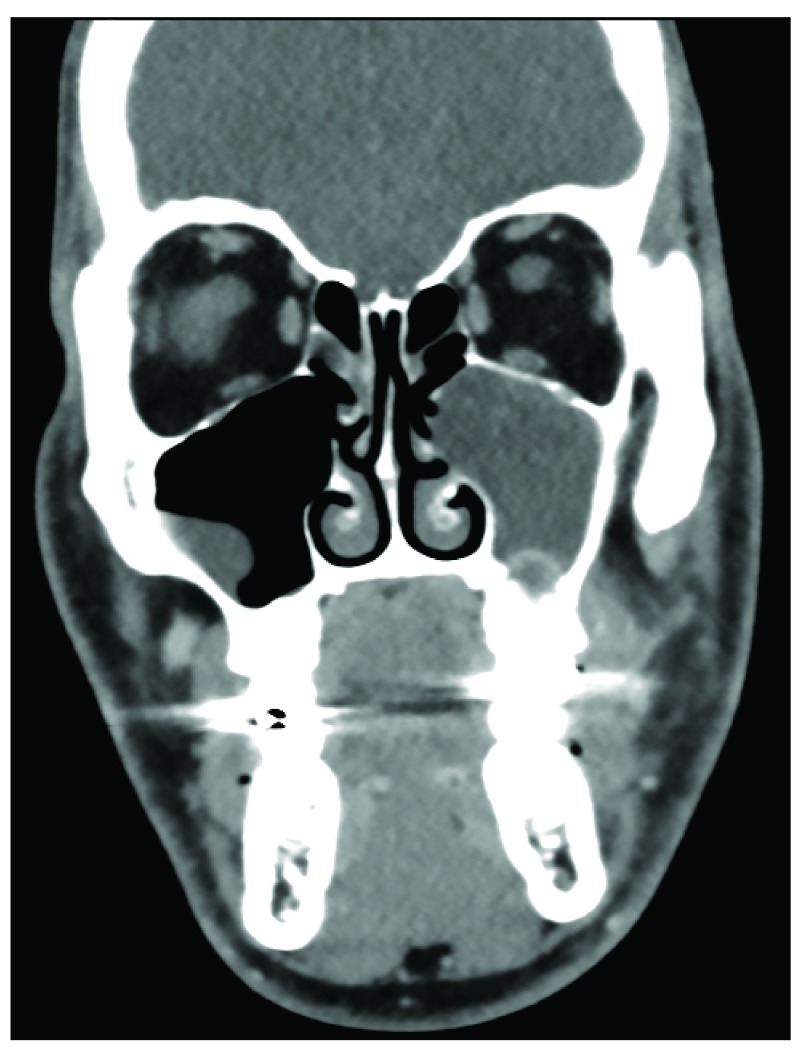
Odontogenic sinusitis. Coronal CT scan demonstrating a periapical lucency extending from tooth #14, resulting in localized left maxillary odontogenic sinusitis.

**Figure 3.**
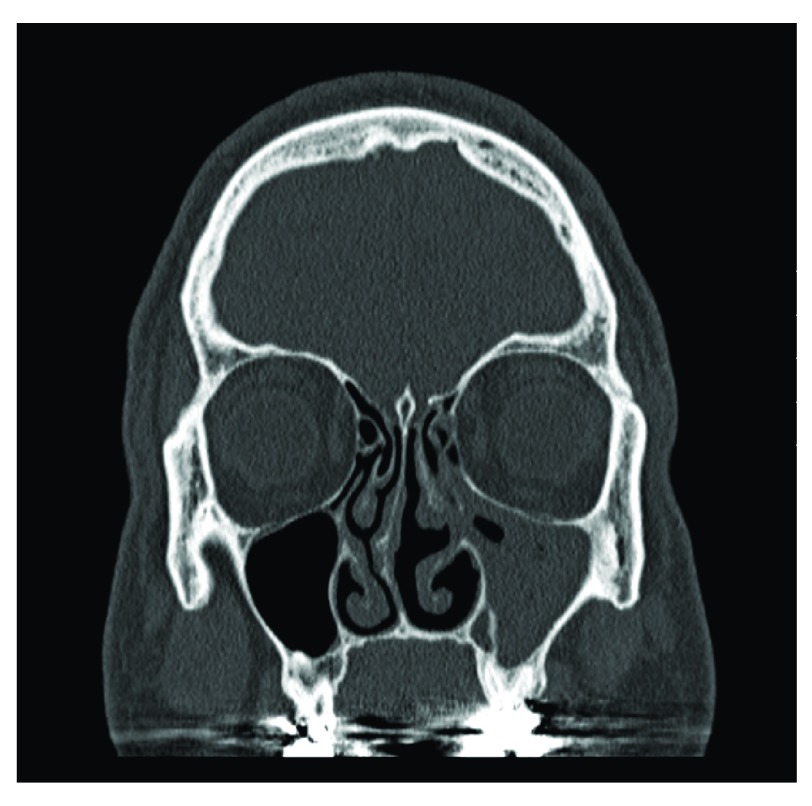
Chronic rhinosinusitis without nasal polyps (CRSsNP). Coronal CT scan of CRSsNP limited to the left maxillary and anterior ethmoid sinuses secondary to a point of obstruction within the left ostiomeatal complex.

Alternatively, the pathophysiology behind some CRSsNP and many RARS patients is more often associated with an anatomic abnormality or localized obstruction rather than a pervasive inflammatory process ^55, 56^. For example, odontogenic sinusitis accounts for approximately 10–12% of RARS and typically presents when a dental abscess or periodontal disease infiltrates the maxillary sinus, resulting in localized sinusitis ( Figure 2) ^57^. By relieving these local obstructions, such as the infected tooth, or other anatomic abnormalities, such as concha bullosa, infraorbital ethmoid cells (Haller cells), and accessory ostia, the disease process can often be halted with minimal surgical intervention. A recent study by Costa *et al*. showed that abnormal anatomy was significantly more common in patients with RARS compared to controls ^58^. Additionally, directed surgery to correct these anatomic abnormalities has been correlated with improvements in symptom scores and shortened operative times ^8, 9, 48, 55^. Certain CRSsNP patients may also benefit from directed surgical intervention depending on the extent of their disease. Figure 3 shows a CT of a patient with CRSsNP limited to his left maxillary and anterior ethmoid cells. Therefore, he underwent a unilateral maxillary antrostomy and anterior ethmoidectomy as the disease appeared localized to a specific point of obstruction in his left ostiomeatal complex. This minimally invasive procedure effectively and efficiently alleviated his symptoms and his CRSsNP.

Balloon dilation of sinus ostia is another surgical technique/tool which has become increasingly popular over the past decade for the treatment of RARS and CRSsNP. This procedure works by dilating the ostia to the maxillary, frontal, and/or sphenoid sinuses, which, in turn, allows for improved nasal irrigations and topical drug delivery to these specific sinuses. Additionally, this technique has the advantage of being able to be performed in the clinic as well as the operating room, possibly obviating the need for general anesthesia. Thus far, multiple studies have shown comparable efficacy to FESS with regard to ostial patency at 1 year, improved symptom scores, reduction in recurrent sinusitis episodes, and improvement in work productivity ^59– 61^. However, many experts agree that this technology should primarily be utilized in a select cohort of patients based on their phenotype, anatomy, and co-morbidities.

Overall, the extent of operative intervention varies greatly from patient to patient with the underlying pathophysiology often playing a significant role. New technological advances along with further understanding of the disease process will likely direct the extent of intraoperative intervention in the future.

## Conclusion

Over the past decade, several factors have become inherent to the successful surgical management of rhinosinusitis. One of the most important aspects appears to be a thorough understanding of the phenotype and, at times, the genotype or endotype of the patient. This plays a role in the perioperative medical management utilized as well as the extent of surgical intervention. It can also allow physicians to more accurately counsel the patient on the likelihood of symptom improvement, the extent of post-operative management, or the chance of disease recurrence/surgical revision.

In this review, we found that CRSwNP patients appear to benefit more from surgical intervention when compared to CRSsNP patients. Additionally, significant evidence exists to support the utilization of perioperative steroids, post-operative antibiotics, and more extensive initial surgical intervention in CRSwNP patients. Alternatively, studies show that CRSsNP and RARS patients are more likely to benefit from correction of any anatomic abnormalities and post-operative antibiotics and topical steroids.

Currently, there are still several gaps in knowledge regarding the optimal surgical management of rhinosinusitis. Specifically, more high-quality randomized controlled trials are needed to examine the effects of perioperative medical management on different CRS subcategories. Additionally, further evaluation into how various CRS endotypes, phenotypes, and genotypes play a role in our ability to predict successful surgical outcomes needs to be undertaken. With these promising advances, we may be able to significantly increase our surgical success rates and improve the quality of life of patients with rhinosinusitis.
